# Circ-RNF13, as an oncogene, regulates malignant progression of HBV-associated hepatocellular carcinoma cells and HBV infection through ceRNA pathway of circ-RNF13/miR-424-5p/TGIF2

**DOI:** 10.17305/bjbms.2020.5266

**Published:** 2021-10

**Authors:** Yan Chen, Shuhua Li, Yinbin Wei, Zhihong Xu, Xiongfei Wu

**Affiliations:** Department of Infectious Diseases, People’s Hospital of Hanchuan, Hanchuan, Hubei, China

**Keywords:** Circ-RNF13, miR-424-5p, TGIF2, HCC, HBV

## Abstract

Circular RNA RNF13 (circ-RNF13; ID: hsa_circ_0067717) is newly identified to be abnormally upregulated in hepatitis B virus (HBV)-associated hepatocellular carcinoma (HCC) patients. However, its role and mechanism remain to be further annotated. First of all, real-time quantitative PCR (RT-qPCR) was utilized to examine RNA expression, and circ-RNF13 was upregulated in HBV-infected human HCC tissues and HBV-expressing cells (Huh7-HBV and Hep3B-HBV), accompanied with TGFβ-induced factor homeobox 2 (TGIF2) upregulation and microRNA (miR)-424-5p downregulation. Loss-of-functional experiments were performed using MTS assay, colony formation assay, flow cytometry, enzyme-linked immunosorbent assay, transwell assay, and xenograft tumor model. As a result, blocking circ-RNF13 enhanced the apoptosis rate of Huh7-HBV and Hep3B-HBV cells, but inhibited cell proliferation, colony formation, migration, and invasion *in vitro*, along with suppressed tumor growth *in vivo*. Besides, RT-qPCR data showed that HBV DNA copies and levels of hepatitis B surface antigen (HBsAg) and hepatitis B e antigen (HBeAg) were diminished by circ-RNF13 knockdown in Huh7-HBV and Hep3B-HBV cells. Mechanistically, circ-RNF13 and TGIF2 could directly interacting with miR-424-5p according to dual-luciferase reporter assay, suggesting that circ-RNF13 and TGIF2 served as competing endogenous RNAs (ceRNAs) for miR-424-5p. Functionally, overexpressing miR-424-5p mimicked and silencing miR-424-5p counteracted the effects of circ-RNF13 depletion in HBV-expressing HCC cells *in vitro*; TGIF2 restoration partially abrogated the role of miR-424-5p upregulation. In conclusion, circ-RNF13 might sponge miR-424-5p to suppress HBV-associated HCC cells malignant progression and HBV infection by regulating TGIF2, providing a novel insight into the occurrence and treatment of HBV-associated HCC.

## INTRODUCTION

Hepatocellular carcinoma (HCC), the overwhelming primary tumor in liver, is renowned as the third leading cause of cancer-related death globally [1]. In china, hepatitis B virus (HBV) infection is a predominant etiology for HCC; chronic hepatitis B (CHB) happens in more than 50% of liver cancers, and accounts for about 80% of virus-associated HCC cases [2]. In the treatment of HBV-related HCC, antiviral therapy has gained widespread attention, and universal vaccination effectively decreases the rate of hepatitis B carriage and slows down the development of HCC in the younger generation in Taiwan [3]. Nowadays, non-viral therapy is emerging as novel hope to therapeutically intervene in the risk and development of HCC tumors [4]. Moreover, great advances in sequencing allows brand-new molecular landscape of HCC progression to be delineated [5], helping to develop novel approaches for effective therapy.

Aberrant expression of regulatory RNAs has been evidenced to contribute to the occurrence and development of HCC with HBV infection [6]. Circular RNAs (circRNAs) are covalently closed RNAs with a stable structure and gene regulation activity. The genome-wide circRNA expression patterns are altered in HBV-related HCC tissue and plasma [7, 8]. Moreover, several circRNAs have been suggested to be circulating fingerprints for predicting the occurrence of HCC with HBV infection [8, 9]. The circ-RNF13 (hsa_circ_0067717, also hsa_circRNA_103489) is one of the five upregulated circRNAs in HBV-associated HCC tissues according to microarray data [10]. However, the roles of circ-RNF13 in HBV infection and HCC malignant progression remain to be annotated, as well as its circRNA/microRNA (miRNA) interactions [7].

MiRNAs are small noncoding RNAs with 21-25 nucleotides that are widely present in eukaryotes [11]. In molecular functions, miRNAs regulate gene expression by binding to the miRNA response elements (MREs) in coding domain sequence or 3’ untranslated region (3’UTR) of target messenger RNAs (mRNAs) [12]. The regulatory role of miRNAs in each stage of HBV-related HCC continuum is collated from early HBV infection, chronic inflammation, fibrosis/cirrhosis, and the onset of HCC [13]. According to a microarray-based genome-wide miRNA analysis, serum miR-424-5p is abnormally upregulated in CHB-induced cirrhosis [14]. Moreover, miR-424-5p is one of the top abnormally downregulated miRNAs in HBV-associated HCC patients’ tissues [15]. Additionally, miRNAs have been considered as early promise in liver cancers including HCC [16]. Therefore, we wondered the relationship between circ-RNF13 and miR-424-5p in HCC cells infected with HBV.

In this study, the expression of above two noncoding RNAs was identified in HBV-associated patients, and the roles of them in HBV-expressing HCC cells were explored. Besides, whether TGFβ-induced factor homeobox 2 (TGIF2), a transcriptional repressor [17], could be one downstream functional gene of circ-RNF13/miR-424-5p axis was further confirmed, and the underlying mechanism on regulating malignant cell progression of HBV-expressing HCC cells and HBV infection was also explored.

## MATERIALS AND METHODS

### HBV-associated patients and tissue samples

A total 23 paired tumor tissues and adjacent liver tissues were collected from 23 HCC patients with chronically active HBV in People’s Hospital of Hanchuan. The clinicopathological characteristics of these patients were shown in Table 1. Clinical specimens were obtained after hepatectomy and immediately frozen in liquid nitrogen. Informed consent was obtained from each subject and this study was approved by the Ethics Committee of People’s Hospital of Hanchuan (approval number 2018ys09) and was carried out according to the guidelines of Declaration of Helsinki.

**TABLE 1:**
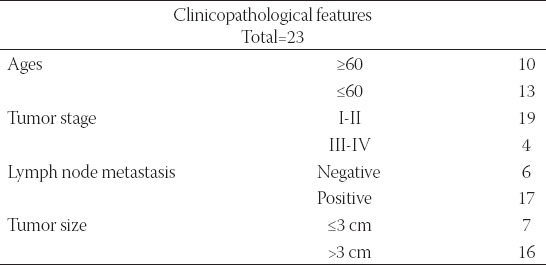
Clinicopathologic features of HBV-associated HCC patients

### HBV-expressing HCC cells and cell transfection

HCC cell line Hep3B (HB-8064) and normal liver epithelial cell line THLE-2 (CRL-2706) were from American Type Culture Collection (Manassas, VA, USA), and Huh7 (GDC0134) was from China Center for Type Culture Collection (Wuhan, China). HBV genome (NC_003977) was cloned in pcDNA3.1(+) (pcDNA; Invitrogen, Carlsbad, CA, USA). Huh7 and Hep3B cells were stably transfected with pcDNA-HBV using Lipofectamine 2000 (Invitrogen) following the manufacturer’s protocol; then 380 mg/L G418 (Promega, Madison, WI, USA) was selected to maintain stable HBV replication for 2 weeks, eventually establishing the HBV-expressing HCC cell lines: Huh7-HBV and Hep3B-HBV. THLE-2, Huh7 and Hep3B cells were cultured in Dulbecco’s modified Eagle’s medium (DMEM; Hyclone, Logan, UT, USA), and Huh7-HBV and Hep3B-HBV cells were cultivated in DMEM supplemented with 380 mg/L G418 (Promega). All cells were incubated in 10% heat-inactivated fetal bovine serum (FBS; Hyclone) and in a humidified atmosphere with 5% CO_2_ at 37°C.

The siRNA against circ-RNF13 (si-circ-RNF13; CGTTGTAAAATCACTGTCGTT) and its negative control (si-NC; UUCUCCGAACGUGUCACGU), miR-424-5p mimic (miR-424-5p; CAGCAGCAAUUCAUGUUUUGAA) and miR-NC (UUUGUACUACACAAAAGUACUG), as well as anti-sense RNA of miR-424-5p (anti-miR-424-5p; UUCAAAACAUGAAUUGCUGCUG) and anti-miR-NC (CAGUACUUUUGUGUAGUACAA) were self-designed and then synthesized by Songon Biotech (Shanghai, China). The overexpression of TGIF2 and circ-RNF13 was implemented in pcDNA (Invitrogen) and pCD-ciR vector (Geneseed, Guangzhou, China), respectively. All these nucleotides were transiently transfected into Huh7-HBV and Hep3B-HBV cells at the indicated concentrations according to the manufacturer’s protocol. The pGPH1/RFP/Neo (GenePharma, Shanghai, China) was used to carry shRNA target circ-RNF13 (sh-circ-RNF13; AACGACAGTGATTTTACAACGAGATGCTG and CGTTGTAAAATCACTGTCGTTAGATGCTG) or sh-NC (TTCTCCGAACGTGTCACGT and ACGTGACACGTTCGGAGAA) prior to neomycin screening for 2 weeks. After transfection for 36 h, the cells were harvested for further analysis.

### RNA isolation and ribonuclease R (RNase R) treatment

Total RNA in tissues and cells was isolated by TRIzol reagent (Invitrogen) according to the manufacturer’s instructions. Cytoplasmic & Nuclear RNA Purification Kit (Norgen Biotek, Thorold, ON, Canada) was utilized to isolate RNA in different subcellular fractions. To identify the stability of circ-RNF13, total RNA (1 μg) was treated with RNase R (3 U; Solarbio, Beijing, China) or diethylpyrocarbonate water for 30 min at 37°C, followed with RNA purification with ethanol precipitation.

### Real-time quantitative PCR (RT-qPCR) and HBV replication analysis

The reverse transcription of RNA into cDNA was fulfilled with High Capacity cDNA Reverse Transcription kit (Applied Biosystems, Foster City, CA, USA), and qPCR was launched using SYBR VR Green Master PCR Mix (Merck, Darmstadt, Germany). The circ-RNF13, RNF13 and TGIF2 were severally amplified with the following primer pairs: circ-RNF13 forward 5’-CCAACGACAGTGATTTTACAACG-3’ and reverse 5’-AGCTGGACAGTCAAGATGGT-3’; RNF13 forward 5’-AGCCACACAAGTCTACACCA-3’ and reverse 5’-ACCAAATCTTGCAGGGAGGT-3’; TGIF2 forward 5’-GACTAACTTGGGTGGGTGGA-3’ and reverse 5’-AACAGATGGGCGACTCAGAA-3’. The miR-424-5p was amplified with primer pair forward 5’-AGCAGCAATTCATGTTTTG-3’ and reverse 5’-GAACATGTCTGCGTATCTC-3’. Glyceraldehyde 3-phosphate dehydrogenase (GAPDH) and RNA U6 small nuclear 1 (U6) were used as endogenous controls and were amplified with primer pairs: GAPDH forward 5’-ATGTTCGTCATGGGTGTGAA-3’ and reverse 5’-CAGTGATGGCATGGACTGT-3’; U6 forward 5’-CTCGCTTCGGCAGCACA-3’ and reverse 5’-AACGCTTCACGAATTTGCGT-3’. The relative expressions were calculated using the 2^-△△Ct^ method.

Cell culture medium was harvested post-transfection for 36 h, and HBV DNA was separated with the Column Viral DNAout kit (TIANDZ, Beijing, China) according to the manufacturer’s instruction. Next, HBV PCR assay reagent II (Qiagen, Valencia, CA, USA) was employed to determine HBV DNA level with the primer pair forward 5’-CCTAGTAGTCAGTTATGTCAAC-3’ and reverse 5’-TCTATAAGCTGGAGTGCGA-3’ [18].

### MTS assay and colony formation assay

Cell proliferation was measured using MTS assay and colony formation assay analyzing cell viability and colony formation. After transfection for 36 h, Huh7-HBV and Hep3B-HBV cells with or without transfection were seeded in 96-well plate at a density of 5 000 cells/well for another cultivation. At 24 h, 48 h and 72 h points after seeding, each well was added with 20 μL MTS reagent from CellTiter 96 AQueous One Solution Cell Proliferation Assays (Promega). The cells were stained with MTS for 2 h with 5% CO_2_ at 37°C, and optical density (OD) value was read on a microplate reader (Bio-Rad, Hercules, CA, USA) by measuring absorbance at 490nm.

Huh7-HBV and Hep3B-HBV cells with transfection or not were seeded in 12-well plate at a density of 150 cells/well. These cells were cultured in complete medium for another 14 days, and fixed with 70% ethanol for 30 min at the room temperature; then, fixed cells were stained with 0.2% crystal violet (Solarbio) for another 30 min. The colonies were visualized and counted under an inverted microscope (Leica, Wetzlar, Germany).

### Flow cytometry (FCM)

Cell apoptosis was evaluated depending on fluorescent labeling of Annexin V-fluorescein isothiocyanate (FITC) and propidium iodide (PI). According to Annexin V-FITC apoptosis kit (Beyotime, Beijing, China), a total of 1×10^5^ cells before and after transfection were harvested, washed, and successively stained with Annexin V-FITC and PI according to manufacturer’s protocol. Apoptosis rate was calculated as the percentage of apoptotic cells (Annexin V-FITC^+^/PI^+/-^; Q1-UR+Q1-LR). For cell cycle analysis, cells were fixed with 70% ethanol and stained with 50 mg/mL PI supplemented with 100 mg/mL RNase (Beyotime) in the dark for 15 min. Then, signals were detected and percentage of cells distributed in G0/G1, S and G2/M phases was determined on flow cytometer (BD Biosciences, Franklin Lakes, NJ, USA) supplemented with Modifit software (BD Biosciences).

### Transwell assays

Transwell chambers (8.0 μm pore size, Corning Costar, Kennebunk, ME, USA) were applied in *in vitro* migration assay. For invasion assay, the upper surface of transwell chambers was needed to be coated with Matrigel (Corning Costar) in advance. Then, 5 × 10^4^ cells in serum-free medium were inoculated in the upper chambers, and completed medium was added in the bottom chambers. These transwell chambers in 24-well plates were incubated with 5% CO_2_ at 37°C for 24 h, and transferred cells on the lower surface of chambers were fixed with 70% ethanol for 30 min prior to crystal violet staining (0.2% for 30 min) at the room temperature. The stained cells were captured under an inverted microscope (Leica) at 100×.

### Enzyme-linked immunosorbent assay (ELISA)

The viral protein hepatitis B surface antigen (HBsAg) and hepatitis B e antigen (HBeAg) in cell culture supernatants of Huh7-HBV and Hep3B-HBV cells were examined by corresponding ELISA kits from Mlbio (Shanghai, China) in line with the instructions of the supplier. OD values were measured at 450 nm.

### Xenograft in nude mice

The animal experiments were complied with the Guide for the Care and Use of Laboratory Animals from NIH (Bethesda, MD, USA), and it was approved by the Ethics Committee of the People’s Hospital of Hanchuan (approval number 2019tn03002). Twenty-eight male BALB/c-nu/nu mice were purchased from Vital River Laboratory Animal Technology Co., Ltd (Beijing, China) and raised for xenograft tumor models. Huh7-HBV and Hep3B-HBV cells stably transfected with sh-circ-RNF13 or sh-NC were subcutaneously injected into the flanks of mice (n=7) at a density of 5 × 10^6^ cells in DMEM supplemented with 10% Matrigel (Corning Costar). The tumor dimensions (mm) were firstly measured using caliper after transplantation for 8 days; then xenograft tumors were monitored every three days. The tumor-bearing mice were euthanatized on the last day (23^th^ from transplantation), and the tumor weight (mg) was measured using electronic balance. Tumor tissues were harvested for total RNA isolation. To draw tumor growth curve, tumor volume (mm^3^) was calculated using the formula 0.5×length×width^2^.

### Dual-luciferase reporter assay

According to starbase v2.0 prediction, the wild-type and mutant-type of circ-RNF13 or TGIF2 3’UTR were inserted into pmirGLO dual-luciferase vector (Promega) to construct vectors carrying WT/MUT-circ-RNF13 or WT/MUT-TGIF2 3’UTR. Huh7-HBV and Hep3B-HBV cells in 96-well plates were co-transfected with above vectors and miR-424-5p or miR-NC. Post-transfection for 36 h, the luciferase signals were read on Dual-Luciferase Reporter Assay System (Promega).

### Protein extraction and western blotting

Total protein in tissues and cells was isolated using RIPA lysis buffer kit (Beyotime), and an aliquot (30 μg) of protein lysate was separated by sodium dodecyl sulphate-polyacrylamide gel electrophoresis. As Follows, proteins were transferred on polyvinylidene difluoride membrane to be probed with primary antibodies against TGIF2 (2D6B6; proteintech, Wuhan, China) or GAPDH (1E6D9; proteintech). The membranes were re-probed with horseradish peroxidase-conjugated Goat Anti-Mouse IgG (H+L) (SA00001-1; proteintech), and signals were detected by enhanced chemiluminescence (Millipore, Bedford, MA, USA). The quantification of band intensity was performed on Image J software (NIH) with normalization with GAPDH.

### Statistical analysis

Assays were individually implemented at least three times for statistical analysis, and the data were shown as mean ± standard deviation with analysis of Student’s *t*-test or one-way analysis of variance followed with Tukey’s post hoc test, *P* values were obtained and signed as * (*p*<0.05), ** (*p*<0.01), *** (*p*<0.001), and **** (*p*<0.0001). Pearson’s correlation analysis was used to assess the correlation among circ-RNF13, miR-424-5p and TGIF2 mRNA expression.

## RESULTS

### Circ-RNF13 was upregulated in HBV-associated human HCC tissues and cells

**FIGURE 1:**
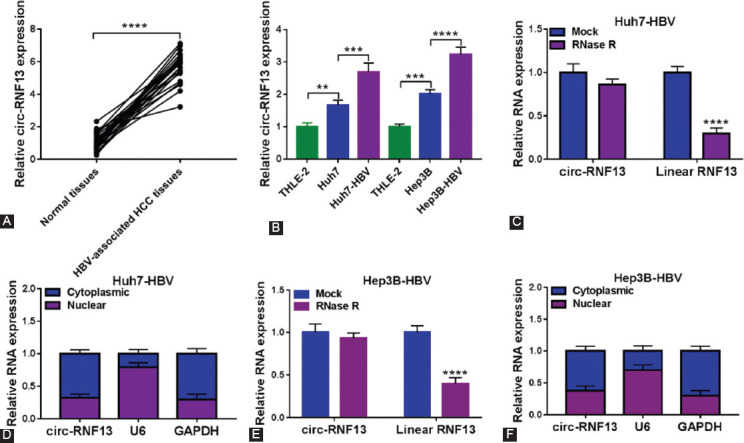
The expression status of circ-RNF13 in HBV-associated HCC tissues and cells. (A) RT-qPCR detected circ-RNF13 expression in paired HBV-associated HCC tissues and adjacent normal tissues from patients (n=23). (B) RT-qPCR detected circ-RNF13 expression in HBV-negative HCC cell lines (Huh7 and Hep3B) and HBV-expressing HCC cell lines (Huh7-HBV and Hep3B-HBV), as well as normal liver epithelial cell line (THLE-2). (C-F) RT-qPCR determined expression of (C, E) circ-RNF13 and linear RNF13 in RNase R-treated RNAs (RNase R group) or diethylpyrocarbonate water-treated RNAs (Mock group), and (D, F) circ-RNF13, GAPDH and U6 in cytoplasmic and nuclear fractions of Huh7-HBV and Hep3B-HBV cells. **, p<0.01, ***, p<0.001 and ****, p<0.0001.

HCC patients with HBV infection were recruited, and expression of circ-RNF13 was upregulated in HBV-associated HCC tumors (n=23) comparing with paired normal tissues (Figure 1A). *In vitro* cells, circ-RNF13 expression level was higher in HBV-negative HCC cells (Huh7 and Hep3B) than normal THLE-2 cells, and more increased in HBV-expressing HCC cells (Huh7-HBV and Hep3B-HBV) (Figure 1B). Thus, circ-RNF13 was an abnormally overexpressed circRNA in HBV-associated HCC tissues and cells. Besides, RNase R could generally digest basically all linear RNAs, and the linear RNF13 expression was significantly reduced whereas circ-RNF13 expression was unaltered with RNase R treatment in Huh7-HBV and Hep3B-HBV cells (Figure 1C and 1E). Paralleled with GAPDH, circ-RNF13 expression in cytoplasmic fraction was about two-fold to that in nuclear fraction, which was different with U6 expression (Figure 1D and 1F). These data indicated circ-RNF13 was a cytoplasmic circRNA with structure stability and was upregulated in HBV-associated HCC samples.

### Blockage of circ-RNF13 suppressed cell proliferation, migration and invasion of HBV-expressing HCC cells *in vitro*, as well as HBV expression and replication

**FIGURE 2:**
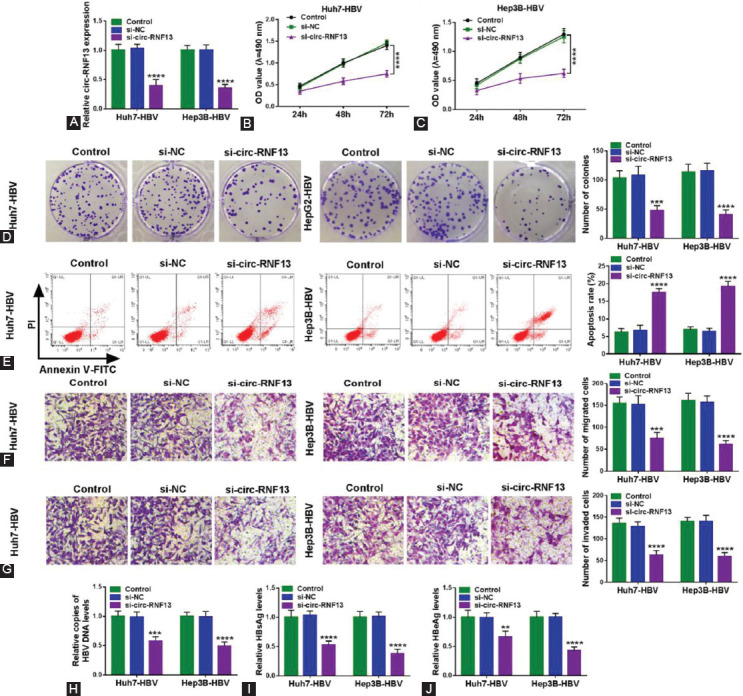
The suppressive role of circ-RNF13 blockage in proliferation, migration, invasion, and HBV expression and replication in HBV-expressing HCC cells in vitro. (A) RT-qPCR measured circ-RNF13 expression in Huh7-HBV and Hep3B-HBV cells without transfection (Control) and with transfection of si-circ-RNF13 or si-NC. (B, C) MTS assay evaluated OD value of above Huh7-HBV and Hep3B-HBV cells after inoculation at 490 nm. (D) Colony formation assay determined number of colonies of above Huh7-HBV and Hep3B-HBV cells. (E) FCM examined apoptosis rate of above Huh7-HBV and Hep3B-HBV cells. (F, G) Transwell assays measured numbers of migrated cells and invaded cells in above Huh7-HBV and Hep3B-HBV cells. (H) RT-qPCR detected copies of HBV DNA level in culture supernatants of above Huh7-HBV and Hep3B-HBV cells. (I, J) ELISA measured levels of viral factors HBsAg and HBeAg in culture supernatants of above Huh7-HBV and Hep3B-HBV cells. **, p<0.01, ***, p<0.001 and ****, p<0.0001.

**FIGURE S1:**
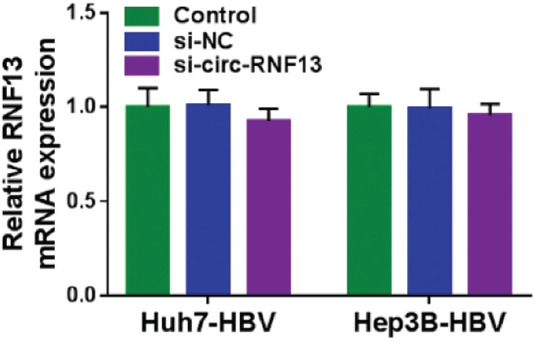
The effect of siRNAs transfection on RNF13 expression. RT-qPCR detected RNF13 mRNA expression in Huh7-HBV and Hep3B-HBV cells without transfection (Control) or transfected with si-NC or si-circ-RNF13.

**FIGURE S2:**
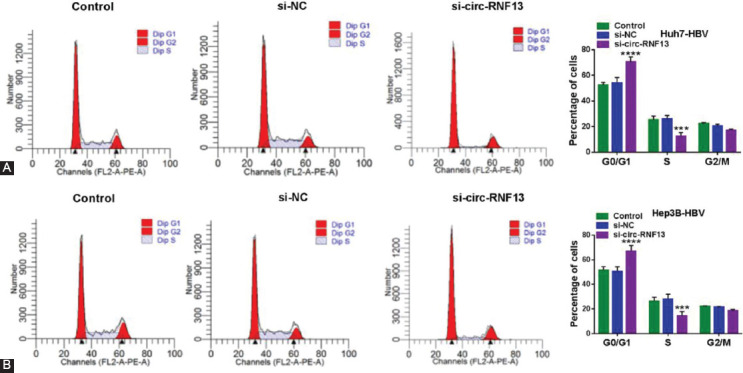
Role of circ-RNF13 knockdown on cell cycle progression of HBV-associated HCC cells. (A, B) FCM examined percentage of cells distributed in G0/G1, S or G2/M phase in Control cells and Huh7-HBV and Hep3B-HBV cells transfected with si-NC or si-circ-RNF13. ***, p<0.001, and ****, p<0.0001.

Loss-of-functional experiments were performed in HBV-expressing HCC cells *in vitro*, and circ-RNF13 expression was specifically silenced in Huh7-HBV and Hep3B-HBV cells after exogenous transfection of si-circ-RNF13 than si-NC (Figure 2A). Meanwhile, si-circ-RNF13 could not knock down the host gene RNF13 expression (Figure S1). MTS assay examined that along with circ-RNF13 downregulation, cell proliferation of Huh7-HBV and Hep3B-HBV cells was lowered, as indicated by smaller OD values during consecutive 3 days (Figure 2B and 2C). Colony formation assay revealed that less number of colonies was formed in Huh7-HBV and Hep3B-HBV cells introduced with si-circ-RNF13 versus si-NC (Figure 2D). As for apoptosis, apoptosis rate was elevated in si-circ-RNF13-transfected Huh7-HBV and Hep3B-HBV cells than si-NC-transfected these cells (Figure 2E), accompanied with more G0/G1 cells and less S cells (Figure S2). Above results demonstrated that silencing circ-RNF13 functioned a proliferation inhibition in HBV-expressing HCC cells *in vitro*. Transwell assays were performed to evaluate cell migration and invasion, and the result was that numbers of migrated cells and invaded cells were reduced due to si-circ-RNF13 administration in comparison with si-NC administration (Figure 2F and 2G). In terms of HBV infection condition, HBV DNA copies in the supernatant of Huh7-HBV and Hep3B-HBV cells were diminished in the presence of si-circ-RNF13 than si-NC (Figure 2H), accompanied with decreased levels of viral factors HBsAg and HBeAg (Figure 2I and 2J). These results suggested a suppressive effect of circ-RNF13 blockage on cell motility and HBV expression and replication in HBV-expressing HCC cells *in vitro*.

### Knockdown of circ-RNF13 retarded tumor growth of HBV-expressing HCC cells *in vivo*

**FIGURE 3:**
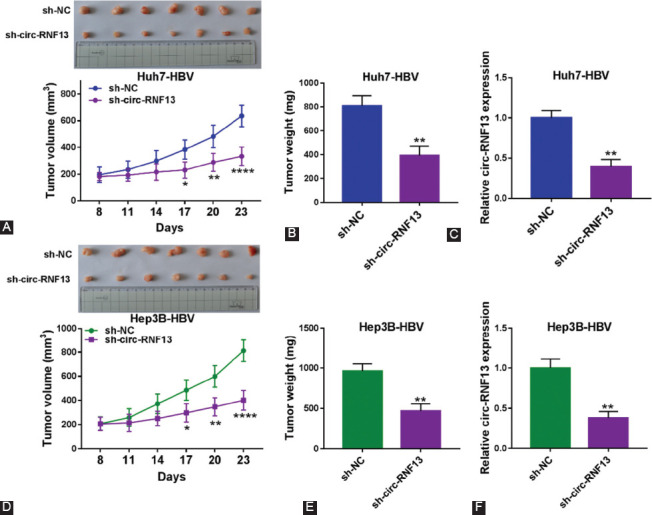
The suppressive role of circ-RNF13 blockage in tumor growth of HBV-expressing HCC cells in vivo. Huh7-HBV and Hep3B-HBV cells stably transfected with sh-circ-RNF13 or sh-NC were transplanted into nude mice (n=7). (A, D) Tumor volume was measured after transplantation for 8 days with every three days and tumor pictures were captured on the 23^th^ day after transplantation. (B, E) Tumor weight was examined on the last day of xenograft assay. (C, F) RT-qPCR detected circ-RNF13 expression in xenograft tumor tissues. *, p<0.05, **, p<0.01, ***, p<0.001, and ****, p<0.0001.

Circ-RNF13 was stably depleted in Huh7-HBV and Hep3B-HBV cells for xenograft experiment in nude mice (n=7/group). Tumor growth was induced in both sh-circ-RNF13 and sh-NC groups, while tumor volume and weight were lowered in sh-circ-RNF13 group (Figure 3A-3B and 3D-3E). Moreover, expression of circ-RNF13 was depressed in xenograft tumors in sh-circ-RNF13 group versus sh-NC group (Figure 3C and 3F). These data showed that circ-RNF13 blockage suppressed growth of HBV-expressing HCC cells *in vivo*.

### Circ-RNF13 served as a sponge for miR-424-5p in HBV-associated HCC cells

**FIGURE 4:**
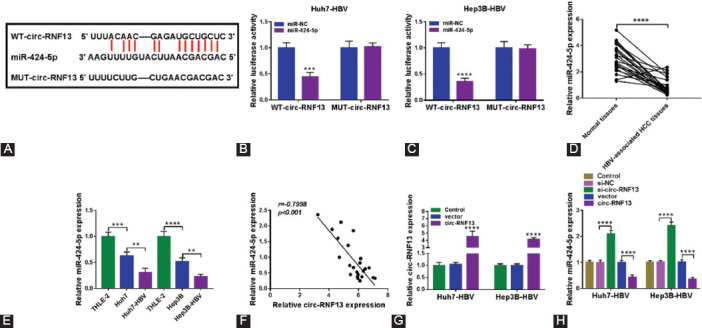
Circ-RNF13 was a sponge for miR-424-5p in HBV-expressing HCC cells. (A) Bioinformatics software predicted sequence alignment among WT-circ-RNF13, miR-424-5p and MUT-circ-RNF13. (B, C) Dual-luciferase activity of WT-circ-RNF13 and MUT-circ-RNF13 in Huh7-HBV and Hep3B-HBV cells transfected with miR-424-5p or miR-NC. (D, E) RT-qPCR detected miR-424-5p expression in tissues from HBV-associated patients (n=23), and cell lines including Huh7, Hep3B, Huh7-HBV, Hep3B-HBV, and THLE-2 cells. (F) Pearson’s correlation coefficient analysis determined the correlation between circ-RNF13 and miR-424-5p expression in HBV-associated HCC tumors. (G, H) RT-qPCR measured circ-RNF13 and miR-424-5p expression in Huh7-HBV and Hep3B-HBV cells without transfection (Control) or transfected with circ-RNF13 overexpression vector (pCD-ciR-circ-RNF13, circ-RNF13), its negative control (pCD-ciR vector, vector), si-NC or si-circ-RNF13. **, p<0.01, ***, p<0.001, and ****, p<0.0001.

**FIGURE S3:**
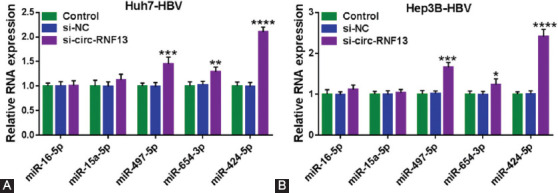
Expression of miRNAs in circ-RNF13-silenced HBV-associated HCC cells. (A, B) RT-qPCR detected expression of miR-16-5p, miR-15a-5p, miR-497-5p, miR-654-3p and miR-424-5p in Control cells and Huh7-HBV and Hep3B-HBV cells transfected with si-NC or si-circ-RNF13. *, p<0.05, **, p<0.01, ***, p<0.001, and ****, p<0.0001.

Functioning as miRNAs sponge had always been one well-documented mechanism for circRNAs in regulating cellular processes. The target miRNAs for circ-RNF13 were predicted on starBase v2.0 (http://starbase/circRNA&mirna/=RNF13/=miR-424-5p), and five computational target miRNAs were preliminarily selected. There were three significantly upregulated miRNAs including miR-497-5p, miR-654-3p and miR-424-5p in circ-RNF13-silenced Huh7-HBV cells, and miR-424-5p was the most sensitive one (Figure S3). Thus, miR-424-5p was selected as the candidate target for circ-RNF13, and MUT-circ-RNF13 was constructed by mutating the complementary sites of miR-424-5p in WT-circ-RNF13 (Figure 4A). The luciferase activity of WT-circ-RNF13 was distinctively attenuated in Huh7-HBV and Hep3B-HBV cells with miR-424-5p overexpression via mimic transfection (Figure 4B and 4C) according to dual-luciferase reporter assay; moreover, MUT-circ-RNF13 luciferase activity failed to be altered with miR-424-5p overexpression. Expression of miR-424-5p was downregulated in both HBV-associated HCC tumor tissues than normal tissues and HBV-positive HCC cells than HBV-negaitve cells (Figure 4D and 4E). Besides, this miRNA expression in this cohort of patients was inversely correlated with circ-RNF13 (Figure 4F). *In vitro*, circ-RNF13 overexpression was induced by its overexpression vector transfection in Huh7-HBV and Hep3B-HBV cells, which was accompanied with lower miR-424-5p expression (Figure 4G and 4H); contrarily, miR-424-5p expression was higher with circ-RNF13 knockdown via siRNA transfection (Figure 4H). These outcomes revealed that miR-424-5p could be regulated by circ-RNF13 via target binding in HBV-associated HCC.

### Silencing miR-424-5p diminished the suppression of circ-RNF13 knockdown in HBV-expressing HCC cells *in vitro*

**FIGURE 5:**
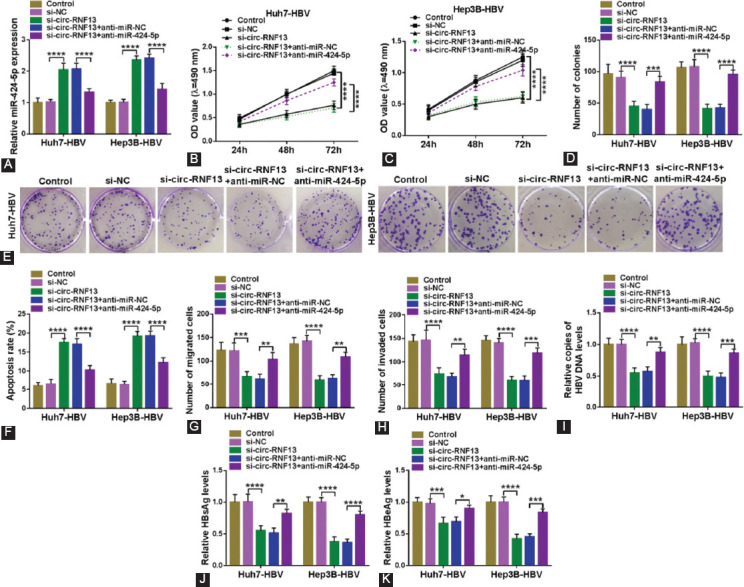
The diminishing effect of blocking miR-424-5p on circ-RNF13 depletion role in HBV-expressing HCC cells in vitro. (A-J) Huh7-HBV and Hep3B-HBV cells were transfected with si-NC alone, si-circ-RNF13 alone or along with anti-miR-424-5p or anti-miR-NC. Post-transfection, (B, C) MTS assay evaluated OD value after inoculation at 490 nm, (D, E) colony formation assay determined number of colonies, (F) FCM examined apoptosis rate, (G, H) transwell assays measured numbers of migrated cells and invaded cells, (I) RT-qPCR detected copies of HBV DNA level in cell culture supernatants, and (J, K) ELISA measured levels of HBsAg and HBeAg in cell culture supernatants. *, p<0.05, **, p<0.01, ***, p<0.001, and ****, p<0.0001.

Functionally, the interactive effect between circ-RNF13 and miR-424-5p was further testified. The si-circ-RNF13 administration led to upregulation of miR-424-5p in Huh7-HBV and Hep3B-HBV cells (Figure 5A); however, circ-RNF13 knockdown-induced miR-424-5p high expression could be silenced with anti-miR-424-5p transfection (Figure 5A). Anti-miR-424-5p administration attenuated the inhibitory effect of circ-RNF13 depletion on cell proliferation and colony formation in Huh7-HBV and Hep3B-HBV cells (Figure 5B-5E). The elevation of apoptosis rate in si-circ-RNF13-transfected both Huh7-HBV and Hep3B-HBV cells was decreased with miR-424-5p depression mediated by anti-miR-424-5p treatment (Figure 5F). Transwell migration and invasion were inhibited in circ-RNF13-silenced Huh7-HBV and Hep3B-HBV cells, and this inhibition was mitigated in the presence of anti-miR-424-5p (Figure 5G and 5H). Blockage of circ-RNF13 restrained HBV infection in Huh7-HBV and Hep3B-HBV cells, which was attenuated by downregulating miR-424-5p via anti-miR-424-5p transfection, as evidenced by the restoration of HBV DNA copies and levels of HBsAg level and HBeAg in the supernatant (Figure 5I-5K). These results indicated an abrogation of miR-424-5p downregulation on circ-RNF13 interference-mediated suppressive role in malignant progression and HBV infection of HBV-expressing HCC cells.

### TGIF2 was a target gene of miR-424-5p in HBV-expressing HCC cells

**FIGURE 6:**
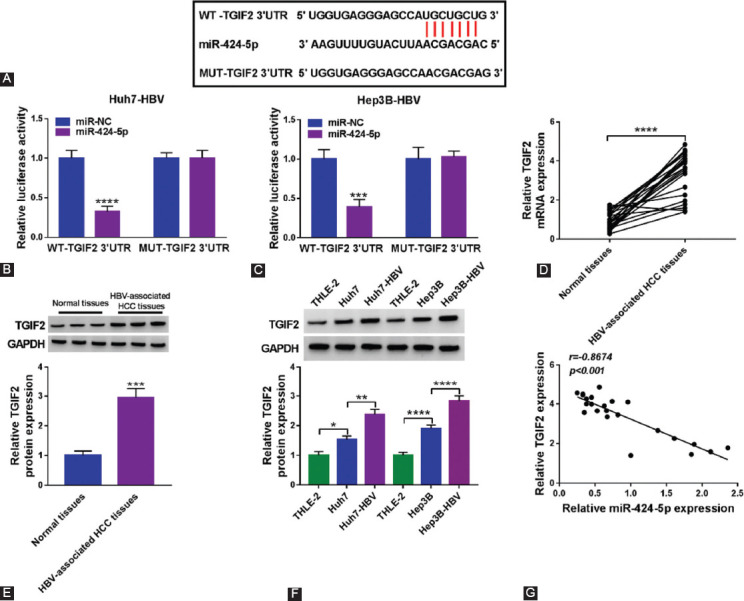
TGIF2 was a target gene of miR-424-5p. (A) Bioinformatics software predicted sequence alignment among miR-424-5p, WT-TGIF2 3’UTR and MUT-TGIF2 3’UTR. (B, C) Dual-luciferase activity of WT-TGIF2 3’UTR and MUT-TGIF2 3’UTR in Huh7-HBV and Hep3B-HBV cells transfected with miR-424-5p or miR-NC. (D, E) RT-qPCR and western blotting detected TGIF2 mRNA and protein expression in tissues from HBV-associated patients. The quantification of band intensity was normalized with GAPDH and compared with the first Normal tissue sample. (F) Western blotting detected TGIF2 protein expression in cell lines including Huh7, Hep3B, Huh7-HBV, Hep3B-HBV, and THLE-2 cells. The quantification of band intensity was normalized with GAPDH and compared with THLE-2 cells. (G) Pearson’s correlation coefficient analysis determined the correlation between TFIF2 mRNA and miR-424-5p expression in HBV-associated HCC tumors. *, p<0.05, **, p<0.01, ***, p<0.001, and ****, p<0.0001.

The novel downstream target gene of miR-424-5p was searched and identified afterwards. StarBase v2.0 (http://starbase/miRNA&mRNA/=hsa-miR-424-5p/=TGIF2) presented the predicted binding sites between miR-424-5p and WT-TGIF2 3’UTR (Figure 6A), and a loss of luciferase activity was observed in Huh7-HBV and Hep3B-HBV cells co-transfected with WT-TGIF2 3’UTR and miR-424-5p (Figure 6B and 6C). TGIF2 mRNA was highly expressed in 23 HBV-associated HCC patients’ tumors (Figure 6D), and its protein expression was higher in tumor tissue from three randomly selected tumors (Figure 6E). *In vitro*, TGIF2 protein expression was upregulated in HCC cells without HBV infection and further increased in HCC cells with HBV infection (Figure 6F). Accidently, there was a negative correlation between miR-424-5p and TGIF2 mRNA expression in these tumor tissues (Figure 6G). These data suggested that TGIF2 was a direct functional gene for miR-424-5p in HBV-expressing HCC.

### Overexpression of miR-424-5p suppressed proliferation, migration, invasion, and HBV expression and replication in HBV-expressing HCC cells *in vitro* via downregulating TGIF2

**FIGURE 7:**
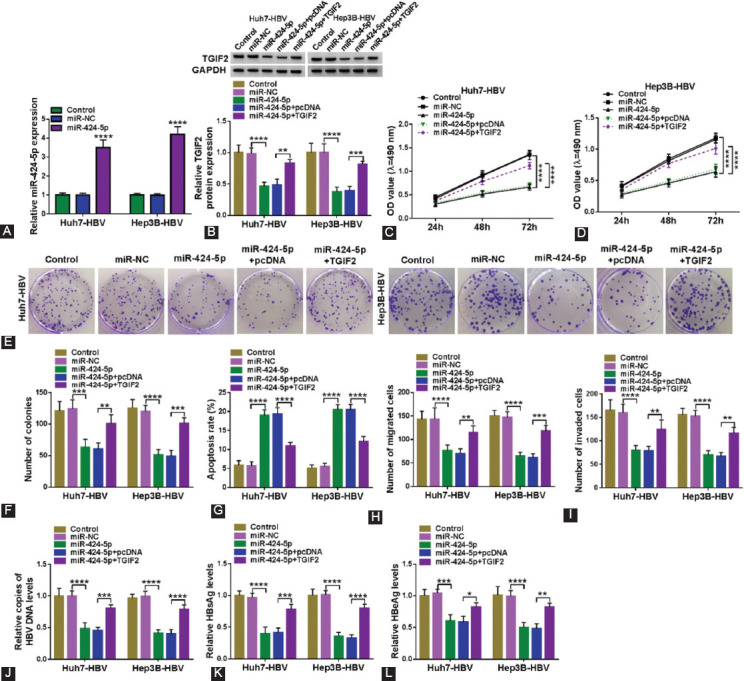
The reciprocal roles of miR-424-5p and TGIF2 in HBV-expressing HCC cells in vitro. (A) RT-qPCR measured miR-424-5p expression in Huh7-HBV and Hep3B-HBV cells transfected with miR-NC or miR-424-5p. (B-L) Huh7-HBV and Hep3B-HBV cells were transfected with miR-NC alone, miR-424-5p alone or along with TGIF2 overexpression vector (pcDNA-TGIF2, TGIF2) or the empty pcDNA vector (pcDNA). (B) Western blot measured TGIF2 protein expression. (C, D) MTS assay evaluated OD value after inoculation at 490 nm. (E, F) Colony formation assay determined number of colonies. (G) FCM examined apoptosis rate, (H, I) transwell assays measured numbers of migrated cells and invaded cells. (J) RT-qPCR detected copies of HBV DNA level in cell culture supernatants. (K, L) ELISA measured levels of HBsAg and HBeAg in cell culture supernatants. *, p<0.05, **, p<0.01, ***, p<0.001, and ****, p<0.0001.

Subsequently, the role of miR-424-5p in HBV-expressing HCC cells was detected, as well as its interaction with TGIF2. MiR-424-5p was abnormally upregulated and TGIF2 was downregulted in Huh7-HBV and Hep3B-HBV cells after exogenous transfection of its mimic (Figure 7A and 7B), and TGIF2 overexpression vector was utilized to improve expression of TGIF`2 in miR-424-5p-transfected cells (Figure 7B). Cell proliferation and colony formation were depressed with miR-424-5p single transfection in Huh7-HBV and Hep3B-HBV cells, which were restored with co-transfection of TGIF2 (Figure 7C-7F). Apoptosis rate was heightened in miR-424-5p-upregulated Huh7-HBV and Hep3B-HBV cells, and this promotion was weakened by improving TGIF2 expression (Figure 7G). Overexpressing miR-424-5p descended transwell migration and invasion of Huh7-HBV and Hep3B-HBV cells, which was rescued due to simultaneously upregulating TGIF2 (Figure 7H and 7I). In the culture supernatant of Huh7-HBV and Hep3B-HBV cells, miR-424-5p upregulation suppressed HBV DNA copies and levels of HBsAg and HBeAg, and this suppression was cancelled by co-upregulation of TGIF2 (Figure 7J-7L). These outcomes demonstrated a suppressive role of miR-424-5p in HBV-associated HCC cells *in vitro* via downregulating TGIF2.

### Circ-RNF13 positively modulated TGIF2 expression by regulating miR-424-5p

**FIGURE 8:**
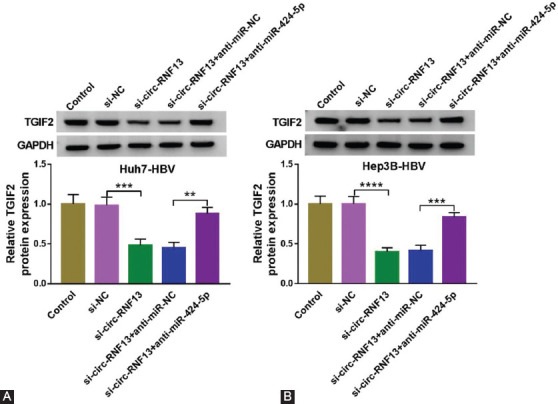
The interactive effect among circ-RNF13, miR-424-5p and TGIF2 in HBV-associated HCC cells in vitro. (A, B) Western blotting tested TGIF2 protein expression in Huh7-HBV and Hep3B-HBV cells transfected with si-NC or si-circ-RNF13, and co-transfected with si-circ-RNF13 and anti-miR-424-5p or anti-miR-NC. **, p<0.01, ***, p<0.001, and ****, p<0.0001.

Above-mentioned results hinted a possible circ-RNF13/miR-424-5p/TGIF2 axis in HBV-expressing HCC cells, and we monitored the interplay among these three informative factors. TGIF2 protein expression was lower in circ-RNF13-silenced Huh7-HBV and Hep3B-HBV cells via siRNA transfection (Figure 8A and 8B), and miR-424-5p downregulation abrogated the regulatory effect of circ-RNF13 knockdown on TGIF2 expression (Figure 8A and 8B). This finding identified a positive regulation of circ-RNF13 on TGIF2 via modulating miR-424-5p in HBV-expressing HCC cells.

## DISCUSSION

Recently, plasma circRNAs including circ_0009582, circ_0037120 and circ_0140117 were discovered to show higher sensitivity and specificity than alpha-fetoprotein (AFP) to distinguish HCC with HBV infection from healthy controls [9]. Moreover, Yu *et al*. declared a panel of three circRNAs (hsa_circ_0000976, hsa_circ_0007750 and hsa_circ_0139897) in the plasma that could diagnose HBV-associated HCC from not only healthy controls, but also CHB and HBV-related liver cirrhosis [8]. In addition, circRNA expression profile in HBV-associated HCC tissues had been uncovered, and the top 5 upreguated circRNAs were hsa_circRNA_104351, hsa_circRNA_102814, circ-RNF13 (hsa_circRNA_103489), hsa_circRNA_102109, and hsa_circRNA_100381, comparing to adjacent non-tumorous tissues [10]. Even though circRNAs dysregulation had been well-documented in HBV-related HCC, the detail role of circRNAs was left to be further annotated.

In this study, circ-RNF13 was clarified to be higher expressed in HCC patients with HBV infection and HBV-expressing cell lines; its expression was mainly distributed in the cytoplasm and was resistant to RNase R. Functionally, silencing circ-RNF13 exerted tumor-suppressive role in HBV-expressing HCC cells (Huh7-HBV and Hep3B-HBV), as indicated by elevated apoptosis and lowered cell proliferation, colony formation, migration and invasion, and tumor growth *in vivo*. Furthermore, HBV DNA expression and levels of HBsAg and HBeAg were also suppressed with circ-RNF13 deficiency, suggesting a suppressive effect of circ-RNF13 knockdown on HBV expression and replication. This study might be the first attempt to illuminate the association among circRNA, HCC development and HBV infection.

Since circRNA-miRNA co-expression networks had already been reported [7], miR-424-5p was further identified as a novel target for circ-RNF13 in this present study. MiR-424-5p was downregulated in HBV-related human HCC tumors than paired normal liver tissues, which was consistent with the former investigation [15]. *In vitro*, miR-424-5p might serve as a tumor suppressor in HCC, due to the decrease of miR-424-5p expression in HCC cell lines with/without HBV expression and its suppression on proliferation, migration and invasion of Huh7-HBV and Hep3B-HBV cells. And, these findings were previously demonstrated by Du *et al*. [19], Yang *et al*. [20] and Yu *et al*. [21]. In addition, anoikis resistance and epithelial-mesenchymal transition process in HCC cells were also reversed by ectopic expression of miR-424-5p [22]. Furthermore, miR-424-5p might clinically predict tumor burden and recurrence, as well as patients’ survival [19, 23, 24]. In mechanism, on one hand, miR-424-5p targeted and regulated functional genes [19, 21, 22]; on the other, server direct regulators of miR-424-5p had also been discovered, including lncRNAs LINC00511 and DLX6-AS1 [25, 26]. Here, we confirmed a novel upstream regulator (circ-RNF13) and a novel downstream effector (TGIF2) of miR-424-5p, and this study might be the pioneer to illuminate miR-424-5p role in HBV-associated HCC cells.

TGIF2 together with TGIF1 repressed gene expression by directly binding to DNA or interacting with TGFβ-responsive SMADs [27]. In liver, TGIF2 seemed to play a multiple role. For example, hepatocytes-expressing TGIF2 was associated with the stepwise reprogramming to a pancreatic progenitor-like phenotype [28]. TGIF2 overexpression affected hepatic lipid metabolism in human hepatoma cells [29], and its upregulation was discovered in HBV-related HCC cells [30]. By the way, we noticed a higher level of TGIF2 in human tissues infected by HBV. Notably, TGIF2 inhibition rendered the inactivation of HBV and the inhibition of proliferation in HBV-expressing HCC cells no matter with overexpression of miR-34c [30] or miR-424-5p (this study). However, the research describing the crosstalk among HCC development, HBV infection and miR-424-5p was still limited and needed to be widely investigated.

High serum HBsAg and HBeAg implied the stable HBV infection [31]. HBsAg quantification was applicable to the treatment of CHB, and high serum HBsAg might predict high risk of HCC and poor outcome of pegylated interferon treatment [32]. However, HBsAg clearance or seroconversion might occur in some patients, which happened only after HBeAg seroconversion [33]. Moreover, HBV DNA could integrated into host genome, and its replication was used for staging and treatment monitoring, as described in international scientific guidelines [34]. Here, circ-RNF13 upregulation and miR-424-5p downregulation were allied with HBV replication and expression in HCC.

In conclusion, circ-RNF13 was an oncogenic in HBV-associated HCC, and blocking circ-RNF13 functioned anticancer role and antiviral role in HCC with HBV infection through regulating miR-424-5p/TGIF2 axis. This study might provide a novel therapeutic target for hepatocarcinogenesis associated with HBV infection.
